# *RSC Advances* Editorial: retraction of falsified manuscripts

**DOI:** 10.1039/d1ra90009a

**Published:** 2021-01-20

As a journal it is our responsibility to uphold the integrity of the scientific record, and this is something we take very seriously. Over the next few weeks, we will be retracting 68 articles published in *RSC Advances*.^1^ These retractions are on the basis of what we believe to be the systematic production of falsified research. Such manipulation of the publication system has been covered widely in the media during 2020,^2,3^ and, while we are not the only journal or publisher to have been affected, this has prompted us to stringently review our processes to ensure that, as far as possible, such papers cannot make it through to publication in the future. We have chosen to publish this editorial in order to be as transparent as possible about the situation, and hope that this will encourage other publishers to take the same approach.

Over the course of 2020 we carried out an extensive investigation into a number of published papers. These papers came to our attention through an update to the ScholarOne system that generated an alert linking many papers from different authors. We began investigating papers that triggered this alert, working with independent image integrity and scientific experts, and consulting with other publishers who are also affected. We identified common features across these papers, as well as instances of image duplication and manipulation to varying degrees. The papers cover similar topics, usually related to manipulation of proteins or other biomolecules to target specific genes or cellular processes for beneficial medicinal effects. Many of these papers are written with very similar structures or templates, despite having no common authors. These papers often appear to be legitimate when viewed in isolation, and many of the concerning features only come to light when comparing features across many papers, making them very difficult for individual editors or reviewers to detect.

*RSC Advances* has always stood for high levels of ethical publishing behaviour, and we are therefore disappointed that our values have been systematically attacked. The size of our journal and breadth of our scope make us a particular target for this type of manipulation, but that does not excuse us from taking responsibility. This is a serious breach of our ethical policies, and as members of the Committee on Publication Ethics (COPE), we have followed their guidelines during our investigation. The thoroughness of our investigation highlights our determination to uphold the highest ethical standards in our journal and we will continue to leave no stone unturned, to ensure there are no more papers linked to this investigation published in our journal. Further information on the individual circumstances for each paper will be included in the retraction notice for that paper.

## Our future actions

As a journal committed to upholding the highest standards of peer review and ethical publishing we are taking a number of actions in order to strengthen our defences against organised and systematic attempts to subvert the publication process.

### Enhanced screening of papers

We have already implemented enhanced screening of papers at the initial stages of assessment, using knowledge gained from this investigation to identify submissions of concern and to reject them. We are also trialling image manipulation software at the initial screening stages that will help to identify instances where images have been altered.

### Improved data requirements

We have implemented stricter data requirements for papers featuring western blots and other types of electrophoresis, requiring authors to provide more complete data to support their conclusions. We are also reviewing the data requirements across the rest of the journal’s scope and making updates where necessary. We will be asking reviewers to specifically comment on whether the data provided with a manuscript meets our new stricter guidelines.

### Training for editors

We will continue to work with our associate editors to ensure they are equipped with the knowledge to be able to identify concerning submissions and take necessary action. We have learned a lot about how to identify suspect papers and what steps we can take to ensure the validity of a submission. We will make sure that all of our associate editors continue to stay informed of any new developments in this area and continue to share learning.

### Recruitment of reviewers

During 2020 we have been working to refresh the *RSC Advances* reviewer panel, ensuring that it fully represents our scope and the diversity of our scientific community, and provides opportunities for early career researchers to gain valuable skills by acting as reviewers. We will continue to recruit new reviewers during 2021 and aim to specifically appoint reviewers with expertise in data and image manipulation, further supporting our associate editors in identifying suspect submissions. We stress that individual reviewers could not be expected to spot individual cases of this type of fraud, but working collaboratively we believe that editors, referees, and journal staff can achieve this.

### Reviewing our scope

While *RSC Advances* is a broad open access journal and these papers were not explicitly outside of our scientific scope, we have taken this opportunity to fully review and refine that scope, noting that, first and foremost, the journal exists to represent the chemistry community. Our clarified scope states that “*RSC Advances* papers should provide an insight that advances the chemistry field. Papers that contain little or no chemistry and are not considered to be of interest or relevance to the chemistry community are not within the scope of the journal.” This clarification will benefit the chemistry community, ensuring that *RSC Advances* only publishes the most relevant content.

Finally, we would like to apologise to our community for the original publication of these papers. While it appears that there has been a systematic effort to dishonestly subvert the publication process, our procedures should have been tighter and we are sorry that we did not reject these papers before publication. We believe the actions we have already taken, along with those planned during 2021, will demonstrate our commitment to remaining a trusted, high quality, open access chemistry journal.

We wish all of our readers and authors a happy and healthy 2021.

Laura Fisher

Executive Editor, *RSC Advances*

Russell Cox

Editor-in-Chief, *RSC Advances*

## Supplementary Material
